# Associations of 24-Hour Movement Behavior with Depressive Symptoms and Anxiety in Children: Cross-Sectional Findings from a Chinese Sample

**DOI:** 10.3390/healthcare9111532

**Published:** 2021-11-10

**Authors:** Shenghua Lu, Boris Cheval, Qian Yu, Md Mahbub Hossain, Si-Tong Chen, Alyx Taylor, Ran Bao, Scott Doig, Jinming Li, Ting Wang, Zhe Yan, Jin Kuang, Can Jiao, Liye Zou

**Affiliations:** 1 College of Sports Science, Jishou University, Jishou 416000, China; lushenghua6666@126.com; 2 Hunan Academy of Education Sciences, Changsha 225002, China; 3 Swiss Center for Affective Sciences, University of Geneva, 1211 Geneva, Switzerland; boris.cheval@gmail.com; 4 Laboratory for the Study of Emotion Elicitation and Expression (E3Lab), Department of Psychology, University of Geneva, 1211 Geneva, Switzerland; 5 Institute of KEEP Collaborative Innovation, Shenzhen University, Shenzhen 518060, China; yuqianmiss@163.com (Q.Y.); jinmingli1999@gmail.com (J.L.); janewang10142021@163.com (T.W.); zoeyan0610@163.com (Z.Y.); dennyppg89@gmail.com (J.K.); liyezou123@gmail.com (L.Z.); 6 Exercise Psychophysiology Laboratory, School of Psychology, Shenzhen University, Shenzhen 518060, China; 7 Department of Health Promotion and Community Health Sciences, School of Public Health, Texas A&M University, College Station, TX 77843, USA; mhossain@tamu.edu; 8 Institute for Health and Sport, Victoria University, Melbourne, VIC 8001, Australia; sitong.chen@live.vu.edu.au; 9 School of Rehabilitation, Sport and Psychology, AECC University College, Bournemouth BH5 2DF, UK; ataylor@aecc.ac.uk; 10 School of Physical Education and Sport Training, Shanghai University of Sport, Shanghai 200438, China; baoryan1955@gmail.com; 11 Department of Physical Education, Limestone University, Gaffney, SC 29340, USA; srdoig@limestone.edu

**Keywords:** movement behaviors, depression, anxiety, primary school students

## Abstract

This study examined the associations between adherence to 24-hour movement behavior guidelines (24-HMB) and the mental-health-related outcomes of depressive symptoms and anxiety in Chinese children. Data on movement behavior from 5357 children (4th and 5th grades), including physical activity, recreational screen time and sleep, were self-reported using the Health Behavior School-Aged Children Survey. Depressive symptoms and anxiety were self-reported using the Chinese version of the nine-item Patient Health Questionnaire and the Generalized Anxiety Disorder Scale, respectively. Depressive symptoms and anxiety were treated as categorical variables. Only 3.2% of the participants met physical activity, screen time, and sleep 24-HMB guidelines. Ordinal logistic regressions showed that, compared with participants who met the 24-HMB guidelines, participants who met none (odds ratio (OR) = 2.62, 95% CI: 1.76–3.90) or any one of the guidelines (OR = 1.88, 95% CI: 1.27–2.77) had higher odds of depressive symptoms. Similarly, there were higher odds of anxiety in participants who met none (OR = 2.32, 95% CI: 1.45–3.70) or any one of the recommendations (OR = 1.62, 95% CI: 1.03–2.57) compared with participants who met all the 24-HMB guidelines. Meeting the 24-HMB guidelines is associated with better mental-health-related outcomes in Chinese children. Because of the low prevalence of Chinese children meeting the 24-HMB recommendations, the present findings highlight the need to encourage children to regularly engage in physical activity, decrease their time spent sitting, and improve their sleep patterns.

## 1. Introduction

Anxiety and depressive symptoms are concerning public health problems, because of their high prevalence and associated adverse health effects [1,2]. Anxiety, defined as excessive worry, and depressive symptoms, defined as persistent unhappiness or sadness, have been recognized as the most prevalent mental illnesses among adolescents [1,2,3]. The Global Disease Burden, the biggest health burden research project across the world, also reported that the global prevalence of depressive symptoms in adolescents is higher relative to decades ago [4], with a prevalence ranging from 7.1–19.4% for European adolescents. Similar values were observed in research on anxiety in adolescents; for example, from 2007 to 2012, anxiety in youth increased by up to 20% [5]. In the US, nationally representative data indicates that 7.1% of 3- to 17-year-old children exhibit anxiety-related problems [1], which are responsible for multiple negative health outcomes [5]. Furthermore, both anxiety and depressive symptoms are associated with an increased risk of engaging in detrimental health behaviors, such as obesity-related behaviors (e.g., excessive screen time and low levels of daily exercise), substance use (e.g., use of cigarettes and alcohol), a lack of social interaction, and even suicidality [6,7,8]. This evidence stimulates an urgent call for effective actions against anxiety and depressive symptoms in adolescents, which can establish a healthier society in the long run.

From the perspective of movement behaviors during a 24-hour period, the three main components, namely physical activity, sedentary behavior (screen time among children and adolescents), and sleep, are closely linked to anxiety and depressive symptoms in children and adolescents [9,10]. Previous studies have demonstrated the independent protective effect of sufficient physical activity, limited screen time, or appropriate sleep on anxiety and depressive symptoms in children and adolescents [11,12,13,14,15]. For example, a Chinese study on adolescents indicated that sufficient physical activity was associated with lower odds of anxiety and depressive symptoms [16]. A cross-sectional study based on Canadian youth indicated that those with higher levels of recreational physical activity had lower risk of depression and anxiety, especially among males, compared with those who did not engage in recreational physical activity [17]. Additional studies have shown that time spent in sedentary behavior may also contribute to an increased risk of developing anxiety and depressive symptoms in youth [18,19]. Finally, reduced sleep duration has also been identified as a contributing factor for anxiety and depression symptoms in adolescents [18,19]. In summary, numerous studies have shown that physical activity, screen time, and sleep are all associated with the mental health of children and adolescents. Yet, these behaviors have been mainly investigated in isolation, with only a handful of studies examining their integrative effects on mental health outcomes.

Recent studies have called upon an integrated and holistic perspective to study well-being rather than examining the multiple determinants of health in isolation [10,20,21]. In particular, as compared with an approach examining each of these three components separately, it has been argued that the investigation of physical activity, screen time, and sleep as a whole could improve their predictive validity of health outcomes [22]. From an applied perspective, this means that changing these three daily-life behaviors together should favor additional health benefits, compared with targeting each behavior in isolation. This change has been exemplified in the release of the Canadian 24-hour Movement (24-HMB) Guidelines for Children and Youth. These guidelines recommend 60 min of moderate to vigorous physical activity, no more than 2 h of screen time, and age-specific sleep duration (9–11 h and 8–10 h are recommended for 6–13-year-olds and 14–17-year-olds, respectively). Such a newly developed research paradigm can help researchers to understand the association of meeting the 24-HMB guidelines with health outcomes in a more comprehensive manner [11,12,23]. Anchored within this integrative approach, recent studies have explored the association of adherence to the 24-HMB guidelines with anxiety and depressive symptoms [23]. For example, Zhu et al. [24] found that meeting all the recommendations of the 24-HMB guidelines was associated with lower odds of anxiety and depressive symptoms among a nationally representative sample of US adolescents. Two similar nationally representative studies from the US and the UK reported a negative association between meeting the 24-HMB guidelines and depressive symptoms in adolescents [25,26]. In summary, this emerging literature supports the positive effects of meeting the 24-HMB guidelines on mental health.

To the best of our knowledge, however, previous studies on the associations between the 24-HMB guidelines and mental health indicators were mainly conducted in western countries. Research findings in western contexts may vary from other contexts owing to different social backgrounds and cultural adaptations. To this end, more studies on 24-HMB guidelines and mental health outcomes are needed to determine whether similar associations of meeting the 24-HMB guidelines with mental health can be generalized in other, less explored, countries such as China. Of note, in China, there is no integrated 24-HMB studies currently. Therefore, investigating the associations between the 24-HMB guidelines and health outcomes among children and adolescents would provide reference for future guidelines in countries such as China. In addition, little is known about associations between specific combinations of recommendations (e.g., meeting none of the guidelines, meeting both physical activity and screen time guidelines, or meeting both physical activity and sleep guidelines) of the 24-HMB guidelines and mental health outcomes. Gaining insight into these specific and detailed associations is essential to design efficient interventions aimed at reducing the burden of mental health problems, including anxiety and depressive symptoms, in children and adolescents.

Collectively, this study was designed to examine the associations of the 24-HMB guidelines with anxiety and depressive symptoms in a sample of Chinese children and adolescents. Specifically, four types of associations were examined: first, the association between meeting the number of recommendations of the 24-HMB guidelines and (1) anxiety and (2) depressive symptoms; second, the association between meeting the combinations of recommendations of the 24-HMB guidelines and (3) anxiety and (4) depressive symptoms.

## 2. Materials and Methods

### 2.1. Study Design and Participants

This cross-sectional study was conducted between March and April 2021. Using a convenient sampling strategy, we invited students at public primary schools to attend this paper-pencil survey. Thirty schools were contacted, of which 26 agreed to participate in this study. Due to the requirements for epidemic prevention, the researchers were unable to enter some schools and failed to negotiate an appropriate investigation time with the schools. Therefore, we failed to arrange for all the schools that were contacted to participate in this study. For each participating school, 4th and 5th graders (10–13 years old) were targeted as study participants. All the students in three randomly selected classes of each grade in the participating school were included. This study excluded children under 10 years old (1st to 3rd graders) owing to their limited ability to respond to the questionnaires in this study. A total of 7083 participants (permitted by their parents or guardians) volunteered to complete the questionnaire, with the assistance of teachers and principals of the participating schools. Data were collected and analyzed anonymously. All invited participating children were informed of the research aim and given instructions prior to signing their consent form. Participants received detailed instructions on how to answer the survey. For this study, after removing cases with incomplete questionnaires and abnormal answers or completion time, 5357 participants who provided valid information on variables were finally included for data analysis. The sample size of our study was calculated using G*Power 3.1 software. Data from previous studies were used to select the parameters for the calculation, including the effect size of 0.2, α at 0.05, and power of 95%, which showed that a sample size of 141 was required per group. Our analytical groups were found to contain more than 141 in all except one combination of criteria, the results for which were treated with caution. The study was approved by the university ethics committee (102772021RT071). All the participants agreed to join the study and parents gave written, informed consent.

### 2.2. Measures

#### 2.2.1. Demographics

Study participants were required to self-report demographic data, including age (years), sex (boy or girl), height (cm), weight (kg), grade (4 or 5), siblings (yes or no), parental education level (middle school or below, high school or occupational school, college or university, master’s or higher degree). Self-reported height and weight were used to calculate the body mass index (BMI) in study participants. According to the World Health Organization’s growth curve, the BMI was categorized into normal, overweight, and obesity.

#### 2.2.2. Physical Activity, Recreational Screen Time, and Sleep Duration

The Health Behavior in School-Aged Children (HBSC) questionnaire was used to measure physical activity [27]. It consists of two items: (1) How many days did you participate in moderate to vigorous physical activity (MVPA) for at least one hour on weekdays in the past week? (0 = none, 1 = 1 day, 2 = 2 day, 3 = 3 days, 4 = 4 days, and 5 = 5 days); (2) How many days did you participate in MVPA for at least one hour on the weekend in the past week? (0 = none, 1 = 1 day, and 2 = 2 days). To avoid potential confusion, a formal definition of MVPA was presented before these two items. MVPA refers to “any kind of physical activity that increased your heart rate and made you breathe hard some of the time (including physical education time, exercise, sports training, and various regular daily activities, such as brisk walking, hiking, and excursion).” A period of 60 min was chosen, because it represents a minimum of daily physical activity recommended for children. Accordingly, this variable allowed measurement of the number of days per week during which the participant met the recommended physical activity guidelines. The reliability was acceptable, and the intraclass correlation coefficient (ICC) of the two items was 0.82 and 0.84, respectively.

The screen time was assessed using the following items derived from the HBSC [27]: (1) How many hours did you spend watching TV or movies in your leisure time on weekdays and weekend days over the past week, respectively? (2) How many hours did you spend playing video games in your leisure time on weekdays and weekend days over the past week, respectively? (3) How many hours did you spend in activities using electronic screen-based devices in leisure time on weekdays and weekend days over the past week, respectively? The responses to these questions were none, about a half-hour, 1 h, 2 h, or 3 h or more. The average daily screen time hours were calculated as follows: Average daily screen time hours = (screen time hours on weekdays × 5 + screen time hours on weekend × 2)/7. According to the Canadian 24-HMB guidelines [21], meeting the screen time recommendation requires a daily screen time less than 2 h per day. The literature confirms the reliability and validity of these items [27]. The reliability was acceptable, and the ICC of the items ranged from 0.73–0.78.

Sleep duration was assessed using the items derived from the HBSC [27]: (1) When do you usually go to bed if you have to go to school the next morning? (2) When do you usually go to bed on weekends or during holidays? (3) When do you usually wake up on school mornings? (4) When do you usually wake up on weekends or during holidays? For the bedtime on weekdays and weekends/holidays, it started at 9:00 p.m. through 2:00 a.m. the next day, with 30 minutes separating each option (no later than 9:00, 9:30, 10:00, etc.). Likewise, with 30-minute intervals, wake-up time slightly varied between weekdays (starting at 5:00 a.m. through 8:00 a.m. or later) and weekends (no later than 7:00 a.m. through 2:00 p.m. or later). Participants’ responses were used to calculate the sleep duration of weekdays and weekends. Then, the average sleep duration per night (hours) was calculated as follows: average sleep duration = (sleep duration per night on weekdays × 5 + sleep duration per night on weekends × 2)/7. Based on the 24-HMB guidelines [21], 9–11 h and 8–10 h are recommended for 6–13-year-olds and 14–17-year-olds, respectively. The reliability was acceptable, and the ICC of the items ranged from 0.64–0.83.

#### 2.2.3. Depressive Symptoms

Depressive symptoms were assessed using the Chinese version of the 9-item Patient Health Questionnaire (PHQ-9). This instrument consists of nine items about experiences of depressive symptoms in the last two weeks. Each item is rated on a four-point Likert scale, ranging from 0 (not at all) to 3 (nearly every day). Total scores range from 0 to 27, with higher scores indicating more severe depressive symptoms. The severity of depressive symptoms can be classified based on PHQ-9 scores: 0–4 (minimal), 5–9 (mild), 10–14 (moderate), 15–19 (moderately severe), and 20–27 (severe) [28]. The psychometric properties of the PHQ-9 have been tested in Chinese children and presented adequate reliability (with a Cronbach’s α coefficient of 0.91) and validity (CFA presented good model fits in this scale: χ^2^ = 364.20, df = 27, *p* < 0.001, CFI = 0.973, TLI = 0.963, RMSEA = 0.069, SRMR = 0.025, indicating good structural validity) [29].

#### 2.2.4. Anxiety

Anxiety was measured using the 7-item Generalized Anxiety Disorder Scale (GAD-7). This instrument consists of seven items, with each item rated on a four-point Likert scale (0 = not at all to 3 = nearly every day). Total scores range from 0 to 21, with a higher score reflecting a greater level of anxiety. The severity of anxiety can be classified as minimal (0–4), mild (5–9), moderate (10–14), and severe (15–21) [30]. The GAD-7 has acceptable specificity and sensitivity for detecting clinically significant symptoms of anxiety in adolescents [31]. The translated GAD-7 has been widely used in Chinese children and adolescents in previous studies [32,33], with a Cronbach’s _α coefficient of 0.93 and a CFA indicating good structural validity with model fits in this scale: χ^2^ = 124.31, *df* = 13, *p* < 0.001, CFI = 0.988, TLI = 0.980, RMSEA = 0.069, SRMR = 0.016.

### 2.3. Statistical Analysis

Statistical analysis was performed using IBM SPSS 24.0. Descriptive statistics were used to report sample characteristics. Specifically, the mean with standard deviation was used to report variables of age and body mass index. For other categorical variables, including sex, grade, siblings, parents’ education, and distribution of meeting the various combinations of the 24-hour movement guidelines, numbers and percentage (%) were used. To investigate the association between 24-hour movement guidelines and each outcome (anxiety or depressive symptoms), two ordinal logistic regression models were developed. The first model concerned the association between the number of recommendations met within the 24-hour movement guidelines and the outcomes. The second model concerned the association between the specific combinations of recommendations the participant met within the 24-hour movement guidelines and the outcomes. In each model, the level of minimal outcomes and meeting none of the 24-hour movement guidelines were treated as reference group. All the models were adjusted for age, BMI, sex, grade, siblings and parents’ education. As our data was nested with cluster structure, we also explored the associations between independents and outcomes by using multilevel mixed models (results can be found in Supplementary Materials). We set *p* < 0.05 as the statistical significance level in this study.

## 3. Results

### 3.1. Sample Characteristics

The final study sample included 5357 Chinese children and adolescents. The mean age was 11.5 years, with a standard deviation (SD) of 0.76 years, and the BMI was 20.2, SD 6.4. More of the participants were boys (55.6%) and had biological siblings (75.5%) (Table 1). Participants’ responses regarding severity of depressive symptoms ranged from minimal (69.1%) to severe (1.2%). Participants’ responses regarding anxiety ranged from minimal to severe, with four-fifths of the participants rating their anxiety as minimal (79.8%). The prevalence of meeting the physical activity, screen time, and sleep guidelines was 11.9%, 65.3%, and 29.1%, respectively. Only 3.2% of participants met all three guidelines within the 24-HMB guidelines, 21.9% of participants met any two guidelines, 52.9% of participants met one guideline, and 21.9% of participants met none of the guidelines. Information on characteristics of participants by sex is shown in Table 1.

### 3.2. Movement Behaviors and Depressive Symptoms

As shown in Table S1, the greater number of meeting recommendations is linked to a lower likelihood of developing depressive symptoms (OR = 0.32). Furthermore, the combination of meeting sleep and screen-time recommendations is linked to the lowest odds (OR = 0.40) of developing depressive symptoms (Table S2). The odds ratio in terms of combination and isolation is presented as follows: MVPA only (OR = 1.1) > Screen time only (OR = 0.73) > Sleep + MVPA (OR = 0.72) > Sleep only (OR = 0.68) > Screen time + MVPA (OR = 0.65) > Sleep + screen time (OR = 0.40). Tables S1 and S2 can be found in Supplementary Materials.

### 3.3. Movement Behaviors and Anxiety

As shown in Table S3, the greater number of meeting recommendations is linked to a lower likelihood of developing symptoms of anxiety (OR = 0.31). Furthermore, the combination of meeting sleep and screen-time recommendations is linked to the lowest odds (OR = 0.35) of developing symptoms of anxiety (Table S4). The odds ratio in terms of combination and isolation is presented as follows: MVPA only (OR = 1.03) > Screen time + MVPA (OR = 0.78) > Sleep + MVPA (OR = 0.72) > Sleep only (OR = 0.68) > Screen time (OR = 0.65) and Sleep only (OR = 0.65). Tables S3 and S4 can be found in Supplementary Materials.

## 4. Discussion

### 4.1. Main Findings

The purpose of this current study was to explore associations between 24-HMB guidelines and two mental health outcomes (anxiety and depressive symptoms) in a sample of 5357 Chinese children. Our results showed that participants who met none or only one recommendation contained in the 24-HMB guidelines have higher odds of being affected by anxiety and depressive symptoms compared with those who met all the three behavior guidelines. These research findings stress the importance of helping children to meet as many movement recommendations as possible, which could be considered a behavioral approach to prevent or treat mental health problems in this age population.

### 4.2. Comparison with Prior Research

Some recent systematic reviews have shown the independent roles of physical activity, screen time, and sleep on mental health outcomes in adolescents [9,34,35,36]. Findings of the present study are consistent with this literature, suggesting that adherence to the 24-HMB guidelines may contribute to mental health benefits in young individuals. For example, Janssen et al. [37] found that those meeting the 24-HMB guidelines had the lowest odds of being affected with mental health issues, along with a positive dose-response relationship (a result that was not observed in the current study). Such difference in the dose-response relationship may be attributed to measurements and participant characteristics. Specifically, the study by Janssen et al. [27] included measures of emotional problems (e.g., feeling low and feeling nervous) as proxy measures of mental health, whereas we used widely recognized scales to measure anxiety and depressive symptoms. Consistent with Zhu et al. [24], our study confirmed that participants meeting none or any one recommendation of the 24-HMB guidelines had higher odds of anxiety and depressive symptoms. This finding seems to be indirectly supported by a previous study by Sampasa-Kanyinga et al. [38], indicating that adolescents having the optimal combination of MVPA, screen time, and sleep had the lowest odds of being depressed or anxious. Given that previous studies mainly focused on westerners [25,39,40], our study can add evidence to the research field of mental health promotion, especially as this is the first time that the beneficial effects of meeting the 24-HMB guidelines on anxiety and depressive symptoms were observed in Chinese children and adolescents. Such findings may further help to refine and update the 24-hour movement guidelines across different cultures.

We found that meeting any two recommendations within the 24-HMB guidelines did not result in significantly higher odds of developing anxiety and depressive symptoms, regardless of the two recommendations in the combination, as compared with those meeting all the three recommendations. This research finding is consistent with previous studies that suggested meeting any two recommendations did not result in significantly higher odds of depressive symptoms [24]. For example, Zhu et al. [24] found that meeting any two recommendations did not result in higher odds of depressive symptoms but did result in higher odds of anxiety. This conflicting finding may be due to different sample characteristics (e.g., age) or the instruments used to assess depressive symptoms and anxiety. The results of our study indicate that meeting any two of the recommendations in the guidelines is as effective as meeting all three recommendations with regard to depressive symptoms and anxiety. These results do not identify which combination of behaviors (e.g., physical activity + screen time, physical activity + sleep, or sleep + screen time) is more effective in preventing anxiety and depressive symptoms. However, it could be suggested that having any two of the healthy movement behaviors might trigger positive effects on the levels of anxiety and depressive symptoms. Further research may determine which specific combinations of behaviors would benefit mental health the most and enable more efficient interventions to be implemented. As it is easier for participants to meet two guidelines than meet all three, it might improve compliance. On the other hand, there are studies indicating that meeting all three recommendations within the 24-hour movement guidelines could lead to additional health benefits [24,37,38,39,40,41]. Therefore, it is recommended that integrated or holistic interventions are used to increase physical activity, limit screen time, and improve sleep simultaneously, as an ideal behavior change approach.

In this study, the prevalence of meeting the 24-HMB guidelines in children and adolescents was relatively low (3.2%). This research finding is consistent with previous studies [42,43,44]. Across the existing literature, young people do not have optimal movement behaviors because of motorized travel and sedentary lifestyles, which is a prevalent and concerning public health problem in the world [45]. Owing to the additional health benefits of 24-HMB guidelines in young people, it is alarming that only very few children and adolescents in our study met the 24-HMB guidelines.

### 4.3. Practical Implications

From the perspective of mental health promotion, encouraging optimal movement behaviors could be an effective way to reduce anxiety and symptoms of depression in children and adolescents. The results from this and previous studies indicate that the aim should be to increase the number of children and adolescents meeting the 24-HMB guidelines (a minimum of 60 min of MVPA per week, no more than 2 h of screen time, and 9–11 h of good quality sleep), with a minimum of two guidelines met.

### 4.4. Strengths and Limitations

To our knowledge, this is one of the first investigations into the possible association between the 24-HMB guidelines and mental health outcomes in young Chinese people, providing practical implications. This studied involved a large sample of Chinese children. Moreover, based on the 24-HMB approach, we measured physical activity, screen time, and sleep in order to determine how meeting recommendations for movement behaviors (and their various/specific combinations) may be associated with two mental-health-related outcomes (i.e., anxiety and depressive symptoms). However, potential limitations should be noted. First, the study sample was recruited using a convenience sampling method, which may not be fully representative of Chinese children, reducing the generalizability of our research findings. Second, given that the data were collected by self-reported measures, the study findings could be influenced by recall and social desirability bias (e.g., height and weight for BMI). Moreover, it should be acknowledged that we cannot explain the mechanism between 24-hour movement guidelines and mental illness outcomes, which should be addressed in the future. Another limitation concerns the relatively small number of possible confounders this study included. This was restricted because we could not require study participants to report more information due to time limitations. This may have influenced the associations estimated between independent variables and outcomes. Future studies should address this study limitation to obtain more robust evidence. Finally, no conclusions about causality could be drawn, as the current study adopted a cross-sectional design. Studies with longitudinal and experimental design are warranted to explore the casual association.

## 5. Conclusions

Our results indicate that children who met none or only one recommendation within the 24-hour movement guidelines are exposed to an increased risk of anxiety and depressive symptoms as compared with those who met all three recommendations of the 24-hour guidelines. The results also indicate that a combination of any two of the recommendations might be as effective in reducing anxiety and symptoms of depression as meeting all three recommendations. Very few children met the 24-hour movement guidelines, which indicates the need for effective actions to change movement behaviors for health promotion. These findings highlight the public health messages of promoting the adoption of healthy movement behaviors by children, which would be a feasible approach for mental health problems. Future studies are required to address our study limitations to gain more robust evidence.

## Figures and Tables

**Table 1:** Participant characteristics of this study.

	Total		Boys		Girls		*p* Value
	n	%	n	%	n	%	
Total	5357	100.0	2976	100.0	2381	100.0	/
Weight status							
Normal	3466	64.7	1925	64.7	1705	71.6	<0.001
Overweight	927	17.2	515	17.3	288	12.1	
Obesity	964	18.0	536	18.0	388	16.3	
Sex							
Boy	2976	55.6	/		/		/
Girl	2381	44.4					
Grade							
4	2770	51.7	1509	50.7	1261	53.0	0.101
5	2587	48.3	1467	49.3	1120	47.0	
Have biological siblings							
Yes	1314	24.5	808	27.2	506	21.3	<0.001
No	4043	75.5	2168	72.8	1875	78.7	
Father education							
Middle school or below	917	17.1	525	17.6	392	16.5	0.001
High school or occupational school	1441	26.9	854	28.7	587	24.7	
College or university	2688	50.2	1425	47.9	1263	53.0	
Master’s or higher	311	5.8	172	5.8	139	5.8	
Mother education							
Middle school or below	1106	20.6	619	20.8	487	20.5	0.027
High school or occupational school	1563	29.2	910	30.6	653	27.4	
College or university	2489	46.5	1332	44.8	1157	48.6	
Master’s or higher	199	3.7	115	3.9	84	3.5	
Met sleep guideline							
No	3796	70.9	2033	68.3	1763	74.0	<0.001
Yes	1561	29.1	943	31.7	618	26.0	
Met screen time guideline							
No	1857	34.7	1089	36.6	768	32.3	0.001
Yes	3500	65.3	1887	63.4	1613	67.7	
Met MVPA guideline							
No	4717	88.1	2543	85.5	2174	91.3	<0.001
Yes	640	11.9	433	14.5	207	8.7	
Met combinations of 24-hour guidelines							
None	1175	21.9	638	21.4	537	22.6	<0.001
Sleep only	471	8.8	308	10.3	163	6.8	
Screen time only	2230	41.6	1135	38.1	1095	46.0	
MVPA only	134	2.5	89	3.0	45	1.9	
Sleep + screen time	841	15.7	462	15.5	379	15.9	
Sleep + MVPA	77	1.4	54	1.8	23	1.0	
Screen time + MVPA	257	4.8	171	5.7	86	3.6	
All	172	3.2	119	4.0	53	2.2	
Met number of 24-hour guidelines							
0	1175	21.9	638	21.4	537	22.6	<0.001
1	2835	52.9	1532	51.5	1303	54.7	
2	1175	21.9	687	23.1	488	20.5	
3	172	3.2	119	4.0	53	2.2	
Severity of depressive symptoms							
Minimal	3700	69.1	2127	71.5	1573	66.1	<0.001
Mild	1149	21.4	612	20.6	537	22.6	
Moderate	322	6.0	152	5.1	170	7.1	
Moderately severe	124	2.3	60	2.0	64	2.7	
Severe	62	1.2	25	0.8	37	1.6	
Severity of anxiety							
Minimal	4277	79.8	2448	82.3	1829	76.8	<0.001
Mild	790	14.7	391	13.1	399	16.8	
Moderate	176	3.3	93	3.1	83	3.5	
Severe	114	2.1	44	1.5	70	2.9	

/ denotes not available. Notes: Abbreviation: MVPA: moderate to vigorous physical activity.

## Data Availability

Data can be requested.

## Supplementary Materials

The following are available online at https://www.mdpi.com/article/10.3390/healthcare9111532/s1. Table S1: The association between number of recommendations meet and depressive symptoms(higher severity). Table S2: The association between specific combinations of recommendations meet and depressive symptoms (higher severity). Table S3: The association between number of recommendations meet and symptoms of anxiety (higher severity). Table S4: The association between specific combinations of recommendations meet and symptoms of anxiety (higher severity).
