# Supplementary material for: Associations of 24-Hour Movement Behavior with Depressive Symptoms and Anxiety in Children: Cross-Sectional Findings from a Chinese Sample
Source: Healthcare (Basel). 2021 Nov 10;9(11):1532. doi: 10.3390/healthcare9111532 (PMC8620023; doi:10.3390/healthcare9111532)
Supplement: Supplementary file 1 [file healthcare-09-01532-s001.zip › healthcare-1358029-supplementary.pdf]

healthcare

MDPI

Table S1. The association between number of recommendations meet and depressive symptoms (higher severity).

Depressive Symptoms				
	Number Met the Recommendations	OR	95% CI	
	3	0.32	0.23	0.46
	2	0.48	0.41	0.55
	1	0.74	0.65	0.83
	0		Ref	
Random Effects				
District		0.01	0.00	0.25
School		0.06	0.03	0.14

OR: odd ratio; CI: confidence interval.; Ref: reference group.

Table S2. The association between specific combinations of recommendations meet and depressive symptoms (higher severity).

Depressive Symptoms				
	Number Met the Recommendations	OR	95% CI	
	All	0.31	0.21	0.44
	Screen time + MVPA	0.65	0.51	0.82
	Sleep + MVPA	0.72	0.48	1.08
	Sleep + screen time	0.40	0.34	0.47
	MVPA only	1.10	0.83	1.47
	Screen time only	0.73	0.64	0.82
	Sleep only	0.68	0.56	0.83
	None		Ref	
Random Effects				
District		0.01	0.00	0.68
School		0.06	0.02	0.15

OR: odd ratio; CI: confidence interval, Ref: reference group.

Table S3. The association between number of recommendations meet and symptoms of anxiety (higher severity).

Anxiety Symptoms				
	Number Met the Recommendations	OR	95% CI	
	3	0.31	0.20	0.48
	2	0.46	0.39	0.55
	1	0.67	0.59	0.76
	0		Ref	
Random Eeffects				
District		0.02	0.00	0.24
School		0.03	0.02	0.16

OR: odd ratio; CI: confidence interval. Ref: reference group.

Table S4. The association between specific combinations of recommendations meet and symptoms of anxiety (higher severity).

	**Anxiety Symptoms**			
	Number Met the Recommendations	OR	95% CI	
	All	0.31	0.20	0.48
	Screen time + MVPA	0.78	0.60	1.01
	Sleep + MVPA	0.72	0.45	1.14
	Sleep + screen time	0.35	0.29	0.43
	MVPA only	1.03	0.75	1.41
	Screen time only	0.65	0.57	0.75
	Sleep only	0.65	0.53	0.81
	None		Ref	
Random Effects				
District		0.02	0.00	0.25
School		0.06	0.02	0.17

OR: odd ratio; CI: confidence interval; Ref: reference group.
